# Novel leiomodin 2b crispant zebrafish as a model for dilated cardiomyopathy

**DOI:** 10.17912/micropub.biology.002079

**Published:** 2026-06-25

**Authors:** Skylie J Antle, Jennifer A Schumacher

**Affiliations:** 1 Department of Biology, Miami University, Oxford, OH, United States; 2 Department of Biological Sciences, Miami University Regionals, Hamilton, OH, United States

## Abstract

Congenital heart disease (CHD) is highly prevalent in live births and requires concise diagnoses and treatment plans. Although there is a vast amount of genetics research in the field of CHD, there are still knowledge gaps regarding the gene networks surrounding heart development. A literature review revealed that mutation in Leiomodin 2 is associated with dilated cardiomyopathy in humans. We selected *lmod2b* for further molecular analysis. In situ hybridization was conducted throughout zebrafish embryonic development to investigate the expression pattern of *lmod2b* . To further understand the function of *lmod2b* , CRISPR-Cas9 gene editing was conducted to look for cardiac abnormalities.

**Figure 1. Expression pattern of lmod2b , heart rate analysis in lmod2b crispants, and CRISPR confirmation gel electrophoresis:** (A-E) shows in situ hybridization expression of *lmod2b* at multiple developmental stages. (A) 21 somites embryo (9/9). Arrowheads indicate adaxial cell expression. (B) 24 hpf embryo (12/12). Arrowhead indicates myocardial tube expression. (C) 26 hpf embryo (12/12). Arrowhead indicates myocardial tube expression. (D) 48 hpf embryo (10/10). Arrowheads indicate atrium and ventricle expression. (E) 72 hpf larva (11/11). Black arrowheads indicate ventricle expression. White arrowhead indicates craniofacial muscle expression. (F) Control larva at 5 dpf. (G) *lmod2b* crispant at 5 dpf. (H) Gel electrophoresis for PCR amplification of *itga4* , and *lmod2b* in exon and 1 exon 2. Lanes 3 and 4 indicate *itga4* controls for uncut genomic regions. Lanes 5 and 6 indicate exon 1 of the genomic region. Lanes 7 and 8 indicate exon 2 of the genomic region. As seen in lanes 6 and 8, crispants showed decreased intensity of bands, showing mosaic gene editing. Arrowhead in lane 8 shows heteroduplex. (I) Heart rate quantification for control and crispant embryos at 4 days post fertilization. Crispants were injected at the 1-cell stage with 1 nl of gRNA cocktail (n= 30 per condition). **** indicates p < 0.0001.

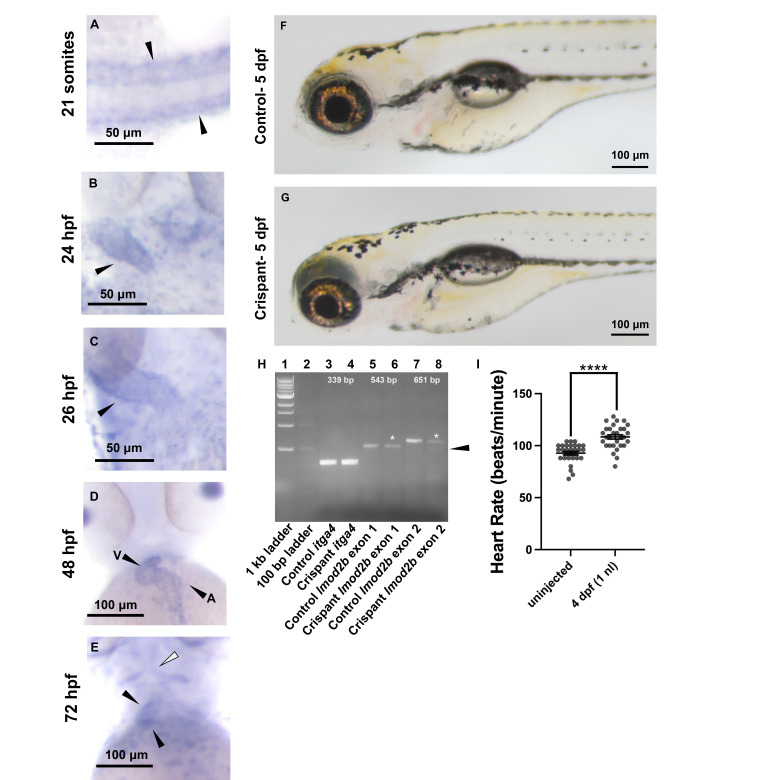



## Description

Dilated cardiomyopathy (DCM) affects about 0.00036% of individuals, and more than 50% of cases have underlying genetic factors (Cleveland Clinic, 2024, https://my.clevelandclinic.org/health/diseases/16932-dilated-cardiomyopathy). In the United States, DCM causes 10,000 deaths and 46,000 hospitalizations annually. However, these statistics may be underestimated due to some patients being asymptomatic. DCM causes weakened heart muscle due to abnormally large chambers of the heart, commonly starting in the left ventricle but can spread to the right ventricle then the atria in severe cases. The myocardium stretches thin, causing it to be weakened (American Heart Association, 2024, https://www.heart.org/en/health-topics/cardiomyopathy/what-is-cardiomyopathy-in-adults/dilated-cardiomyopathy-dcm). Left ventricular dysfunction causes blood to remain in the heart after each contraction, so the heart must compensate to ensure blood flow to the rest of the body. This leads to an overall higher heart rate (HR), known as tachycardia (Shoureshi et al., 2024). Patients often present with chest pain, dizziness or fainting, fatigue, heart palpitations, and dyspnea. These symptoms are manageable if detected early and properly treated (Cleveland Clinic, 2024, https://my.clevelandclinic.org/health/diseases/16932-dilated-cardiomyopathy). A vast number of studies relating to cardiomyopathy have been conducted, however the genetic basis of this condition is still unclear. *LMOD2* is a causal gene of DCM but is less characterized than other DCM causal genes such as *BAG3* , *DES* , *FLNC* , *LNMA* , *MYH7* , *PLN* , *RBM20* , *SCN5A* , *TNNC1* , *TNNT2* , and *TTN* (Jordan et al., 2021). Mutations in LMOD2 that lead to impaired protein production can cause early-onset DCM that is fatal without proper medical intervention (Pappas et al., 2024).

Leiomodin gene family members are expressed in skeletal, smooth and cardiac muscle in adults, as well as during the fetal period (Conley et al., 2001). However, leiomodin 2 (Lmod2) is limited to cardiac and skeletal muscle (Tsukada et al., 2010). These striated muscles use sarcomeres to contract, which requires proper organization of actin and myosin filaments. Lmod2 is an actin nucleator, catalyzing the formation of new actin filaments. Lmod2 is necessary for regulating the length of thin filaments of cardiac muscle (Wu et al., 2017), which is crucial for maintaining the structure of sarcomeres, allowing for proper contractile function. When Lmod2 function is disrupted, abnormalities in thin filament structure can result in reduced cardiac muscle function (Wu et al., 2017).

In avian embryos, *LMOD2* transcripts are expressed after the heart begins beating (Hamburger-Hamilton stage 14), indicating it is not the first actin nucleator involved in thin filament assembly (Tsukada et al., 2010). Overexpression of mCherry-Lmod2 in avian cardiomyocytes resulted in disorganized sarcomere structure and overextension of thin filaments, interfering with the typical striated patterns. In mouse embryos, *Lmod2* transcripts are expressed in the heart at embryonic day 8.5, and in the somites at embryonic day 9.5 (Pappas et al., 2015). *Lmod2* knockout (KO) mouse models were found to have abnormal cardiac contractility, shortened actin-thin filaments, and irregular calcium handling. Lmod2 KO resulted in fatal neonatal DCM causing mice to die between 15 and 33 days after DCM onset. When Lmod2 was restored in *Lmod2* -KO mice at postnatal day 4, before heart defects were detected, thin filament length increased, nearly reaching normal levels (Pappas et al., 2015).

We use zebrafish as a model for cardiac development, as they lay large amounts of transparent embryos that allow for analysis of internal organs. Zebrafish have two-chambered hearts that begin beating at 24 hours post fertilization (hpf), shortly followed by blood flow. The simplicity of the two-chambered heart with rapid development of the cardiovascular system allows for quick data collection and analysis. Conserved signaling pathways and orthology to human disease makes zebrafish an excellent model for developmental processes. Zebrafish are a great model organism for cardiovascular development because they can survive up to five days post fertilization without a heartbeat. This resilience allows insight into the intricacies of the cardiovascular system, making them beneficial when compared to other model organisms. Zebrafish are extremely useful as preclinical models, serving as a gateway to test the effectiveness of new therapeutic drugs that could potentially be used to treat symptoms of cardiomyopathy (Patton et al., 2022). Previous studies have shown that homozygous mutant *lmod2a* -/- zebrafish have irregular heart rhythms and impaired contractility, indicating that *lmod2a* plays an essential role in thin filament regulation (Ye et al., 2025). However, the role of *lmod2b* remains uncharacterized. *lmod2b* is predicted to be involved with actin filament organization, tropomyosin bind activity, muscle contraction, and myofibril assembly in zebrafish.

Using in situ hybridization with the *lmod2b* RNA probe, we saw cardiac expression at 24 hours post fertilization (hpf) which aligns with this gene's predicted expression patterns. We also saw expression in the skeletal muscle structures which aligns with its predicted role in skeletal muscle contraction. Using CRISPR-Cas9 technology, we created a novel zebrafish model by mutating the *lmod2b* gene to further our understanding of DCM. We found no significant pericardial edema or abnormalities in the craniofacial structure when comparing the control group to crispants. However, our findings are consistent with a potential role for *lmod2b* in DCM, as *lmod2b* crispants had higher heart rates compared to the control group.

**Discussion**

In situ hybridization was conducted at multiple developmental stages to analyze expression patterns of *lmod2b* . At 21 somites, *lmod2b* was expressed in adaxial cells of the somites ( Figure 1A ). At 24 and 26 hpf, *lmod2b* was expressed in the myocardial tube ( Figure 1B- C). First detection of *lmod2b* in the myocardial tube at 24 hpf when the heart begins to beat aligns with expression pattern data in avian embryos. At 48 hpf, *lmod2b* was expressed in both the ventricle and atrium ( Figure 1D ). At 72 hpf, *lmod2b* was expressed in the ventricle and craniofacial muscles ( Figure 1E ). As hypothesized, *lmod2b* was expressed in the cardiovascular system during early stages of embryonic development, indicating potential importance for early cardiovascular function. Notable expression in the atrium at 48 hpf but not at 72 hpf aligns with changes in atrial morphology. The atrial wall thins by 72 hpf, likely resulting in weaker expression at 72 hpf that may not be detected by in situ hybridization. Expression in the craniofacial muscles indicates involvement in the skeletal muscle system, which supports its predicted roles in contractile function.

Tg(kdrl:NLS-EGFP) ^ubs1Tg^ embryos were injected with a cocktail of four gRNAs and purified Cas9 protein to mutate *lmod2b* (Blum et al., 2008) *.* Alterations at the *lmod2b* locus were confirmed with PCR amplification of genomic DNA ( Figure 1H ). We found that there was decreased amplification of the *lmod2b* gene in crispant DNA, however, there was still some wild-type DNA present, indicating mosaicism. Uninjected control and *lmod2b* crispant embryos were analyzed daily to check for abnormalities. The overall morphology of the embryo and the heart appeared normal in the crispants ( Figure 1F- G), however heart rate analysis revealed abnormalities. Across multiple experiments, the *lmod2b* crispant zebrafish had higher heart rates than the controls ( Figure 1I ). Crispants injected with 1 nl gRNA mix had a heart rate average of 108.4 beats per minute compared to the control group, which had an average of 92.8 beats per minute (n= 30 per condition). This indicates that *lmod2b* gene mutation resulted in tachycardia, which aligns with symptoms of DCM. We did not find notable abnormalities in blood flow or pericardial edema in *lmod2b* crispants ( Figure 1F- G). This mild crispant phenotype aligns the unmutated DNA still present at the molecular level. Based on the heart rate results, we can infer that there may be subtle defects within the structure of the heart that could be revealed with higher resolution analysis.

Moving forward, we will continue investigating heart morphology of *lmod2b* crispants by conducting MF20 and S46 immunostaining, which mark myosin heavy chains in both chambers and only the atrium, respectively. This could highlight cardiac defects that are not visible under normal stereomicroscopy conditions. Mutation of *lmod2a* and *lmod2b* in zebrafish both result in cardiac abnormalities, which suggests that these genes are not functionally redundant, and each plays a specific role in cardiac function. Based on this observation, we will further investigate the genetic networks by conducting a double knockdown of *lmod2a* / *lmod2b* . We will analyze the double knockdown crispants to continue understanding the molecular mechanisms of DCM. Creating a stable mutant line in the future will allow us to evaluate any defects that occur through adulthood and calculate survival rate.

## Methods

RNA probe synthesis: Total RNA was extracted and purified from 96 hpf wildtype zebrafish embryos. Reverse transcription using Superscript IV (Invitrogen) was then used to create cDNA. Forward and reverse primers were designed to amplify a 957 base pair (nucleotides 281-1237) section of the *lmod2b* coding sequence. Primer pair used is as follows: forward primer sequence 5’-CTCCACCGGTACATTCAGT-3’ and reverse primer sequence 5’-CTTGATTGCGGGTGAGAAGG-3’. PCR product was obtained for cloning with TOPO technology. Directionality of insertion was determined by Sanger sequencing. Plasmid linearization for probe synthesis was conducted using NotI restriction enzyme. SP6 RNA polymerase was used for in-vitro transcription. Digoxigenin labeled antisense RNA probe was precipitated and stored at -80°C.

In situ Hybridization: Zebrafish embryos were fixed at 21 somites, 24 hpf, 26 hpf, 48 hpf, and 72 hpf. Whole mount in situ hybridization was conducted at each developmental stage using *lmod2b* antisense RNA probe as previously described (Oxtoby & Jowett, 1993). Proteinase K treatment (10 mg/ml) was performed for 5 to 25 minutes at room temperature, depending on the stage examined. Samples were incubated in pre-hybridization solution for 2 hours at 65°C, followed by incubation with antisense RNA probes in hybridization solution at 65°C overnight. Pre-hyb/SSC wash series was completed at 65°C in 15 minute increments. Samples were placed in 5% sheep serum blocking solution for 1 hour at room temperature followed by Anti-Digoxigenin-AP Fab fragments antibody solution (1:5000) overnight at 4 °C. Staining was conducted with NBT/BCIP at room temperature until signal was visible. Samples were transferred to 80% glycerol for imaging with a Zeiss SteREO Discovery.V8 microscope equipped with a Zeiss Axiocam 506 color camera.

gRNA synthesis and CRISPR analysis: Oligonucleotide sequences were designed based on Wu et al. (2018) Genome-Scale Lookup Table for Four-Guide Sets. CRISPR sequences selected from this article include: GCGTTCTGGGATCCTTCGAG (PAM site TGG), AGGATTGGATCTACAAGCCC (PAM site AGG), GTACCTCATCAGCCACCGTG (PAM site GGG), and GGGTTGTCATGCTTATACGT (PAM site GGG). PCR products were obtained and purified. *lmod2b* gRNA was synthesized using T7 polymerase. A 1:2 ratio of purified gRNA to Cas9 protein was used to create the injection mix. The final concentration of Cas9 was 500 ng/ul and the final concentration of gRNA was 250 ng/ul. We injected 1 nl into 1-cell Tg(kdrl:NLS-EGFP) ^ubs1Tg^ zebrafish embryos. Embryos were incubated at 28.6°C until heart analysis at 4 days post fertilization (dpf). For heart rate quantification, randomly selected larvae were individually transferred in a minimal amount of liquid into a depression slide containing 160 mg/mL tricaine stock solution. The larva was then incubated for 1 minute before measuring heart rate. Heart rate was quantified visually using a stereomicroscope by counting beats with a tally counter for 15 seconds and multiplying by 4 to be recorded in beats per minute. Each larva was incubated individually in fresh tricaine solution for 1 minute before HR was counted to ensure consistent anesthetic treatment across all samples. Based on experimental set up, the investigator was not blinded to experimental conditions. Analysis of control and crispant embryos was replicated 3 times with n=10 per condition for each trial.

CRISPR confirmation: To confirm editing of the *lmod2b* locus on the molecular level, genomic DNA was extracted from control and crispant embryos at 24 hpf. Zebrafish were anesthetized with tricaine and sorted into PCR tubes. To extract DNA, lysis buffer was prepared with 10 mM Tris HCl pH 7.5, 50 mM KCl, 0.3% Tween-20, 0.3% NP-40, and 1 mM EDTA pH 8. Lysis buffer was added and embryos were incubated for 10 minutes at 95° C. Proteinase K treatment (2 µg/ml) was performed at 55° C for 12 hours followed by enzyme inactivation at 95° C for 10 minutes. After lysate was obtained, genomic DNA was amplified using PCR. Primer pairs based on guide RNA sites were designed for PCR in CHOPCHOP. Primer pair 1 included forward primer sequence 5’-TTTTGCAGCTCTTTCTGATTGA-3’ and reverse primer sequence 5’-TTTTCTGACACCATTGGAAGTG-3’. Primer pair 1 amplified across the genomic region spanning exon 1, where gRNAs 1 and 2 cut (nucleotides 185,713-186,255). Primer pair 2 included forward primer sequence 5’-AAAGGCAGATAGGTGAACCAAA-3’ and reverse primer sequence 5’-AGCTGTGAGGAGAAGAAGTGCT-3’. Primer pair 2 amplified the genomic region spanning exon 2, where gRNAs 3 and 4 cut (nucleotides 184,171-184,821). Primers for *itga4* gene were used as a control for an uncut region of the genome (Schumacher et al., 2020). *itga4* is on chromosome 9 whereas *lmod2b* is on chromosome 4, ensuring there would be no CRISPR cleavage at this site. Gene mutation was confirmed using gel electrophoresis on a 1.5% agarose gel.

## Reagents

Integrated DNA Technologies: Oligonucleotides 

Invitrogen: Superscript IV Reverse Transcriptase Kit

Millipore Sigma: Anti-Digoxigenin-AP Fab fragments

New England Biolabs: PCR & DNA Cleanup Kit 

New England Biolabs: HighScribe T7 kit 

New England Biolabs: RNA clean up kit 

New England Biolabs: *Taq* DNA polymerase

New England Biolabs: NotI restriction endonuclease

PNA Bio: Cas9 Protein 

QIAGEN: QIAprep Spin Miniprep Kit 

ThermoFisher Scientific: TOPO TA cloning kit 

ThermoFisher Scientific: One Shot TOP10 chemically competent *E. Coli*
